# Cervical Screening within HIV Care: Findings from an HIV-Positive Cohort in Ukraine

**DOI:** 10.1371/journal.pone.0034706

**Published:** 2012-04-24

**Authors:** Heather Bailey, Claire Thorne, Igor Semenenko, Ruslan Malyuta, Rostislav Tereschenko, Irina Adeyanova, Elena Kulakovskaya, Lyudmila Ostrovskaya, Liliana Kvasha, Mario Cortina-Borja, Claire L. Townsend

**Affiliations:** 1 MRC Centre of Epidemiology for Child Health, UCL Institute of Child Health, University College London, London, United Kingdom; 2 Perinatal Prevention of AIDS Initiative, Odessa, Ukraine; 3 Odessa Regional Centre for HIV/AIDS, Odessa, Ukraine; 4 Kiev City Centre for HIV/AIDS, Kiev, Ukraine; 5 Donetsk Regional Centre for HIV/AIDS, Donetsk, Ukraine; 6 Mykolaiv Regional Centre for HIV/AIDS, Mykolaiv, Ukraine; 7 Kriviy Rig City Centre for HIV/AIDS, Kriviy Rig, Ukraine; University of Alabama at Birmingham, United States of America

## Abstract

**Introduction:**

HIV-positive women have an increased risk of invasive cervical cancer but cytologic screening is effective in reducing incidence. Little is known about cervical screening coverage or the prevalence of abnormal cytology among HIV-positive women in Ukraine, which has the most severe HIV epidemic in Europe.

**Methods:**

Poisson regression models were fitted to data from 1120 women enrolled at three sites of the Ukraine Cohort Study of HIV-infected Childbearing Women to investigate factors associated with receiving cervical screening as part of HIV care. All women had been diagnosed as HIV-positive before or during their most recent pregnancy. Prevalence of cervical abnormalities (high/low grade squamous intraepithelial lesions) among women who had been screened was estimated, and associated factors explored.

**Results:**

Overall, 30% (337/1120) of women had received a cervical screening test as part of HIV care at study enrolment (median 10 months postpartum), a third (115/334) of whom had been tested >12 months previously. In adjusted analyses, women diagnosed as HIV-positive during (vs before) their most recent pregnancy were significantly less likely to have a screening test reported, on adjusting for other potential risk factors (adjusted prevalence ratio (APR) 0.62, 95% CI 0.51–0.75 *p*<0.01 for 1^st^/2^nd^ trimester diagnosis and APR 0.42, 95% CI 0.28–0.63 *p*<0.01 for 3^rd^ trimester/intrapartum diagnosis). Among those with a cervical screening result reported at any time (including follow-up), 21% (68/325) had a finding of cervical abnormality. In adjusted analyses, Herpes simplex virus 2 seropositivity and a recent diagnosis of bacterial vaginosis were associated with an increased risk of abnormal cervical cytology (APR 1.83 95% CI 1.07–3.11 and APR 3.49 95% CI 2.11–5.76 respectively).

**Conclusions:**

In this high risk population, cervical screening coverage as part of HIV care was low and could be improved by an organised cervical screening programme for HIV-positive women. Bacterial vaginosis testing and treatment may reduce vulnerability to cervical abnormalities.

## Introduction

Ukraine has the highest adult HIV prevalence in Europe, estimated at 1.6% in 2007 [1]. Heterosexual transmission has now overtaken injecting drug use (IDU) as the main mode of HIV acquisition [2], and women account for almost half of those living with HIV in Ukraine [3]. HIV-positive women are at increased risk of acquisition and/or persistence or reactivation of cervical infection with Human papillomavirus (HPV) [4]–[6]. This is due to shared risk factors for acquisition (e.g. multiple sexual partners) and immunosuppression [7], [8]. Cervical abnormalities in HIV-positive women are more likely to be severe, aggressive and resistant to treatment [9], [10], and HIV-positive women have a 5 to 8-fold increased risk of invasive cervical cancer compared with the general population [11], [12].

Regular cytologic screening can effectively reduce the incidence of invasive cervical cancer by up to 80% on a population level [13], [14]. A two to three year screening interval is recommended by British and US guidelines for HIV negative women of childbearing age [15], [16], with more frequent (at least annual) screening for HIV-positive women, given their increased risk of morbidity [17], [18]. Screening during pregnancy is not recommended by UK guidelines [15] because invasive cervical cancer in pregnant women is very uncommon, and treatment for less severe abnormality must be deferred until after delivery [19]. However, antenatal screening may be justified for HIV-positive women given their increased cancer risk. Perinatal care can also be an opportunity to engage marginalised women, who are unlikely to attend for screening at another time, and those who have previously defaulted on follow-up appointments for abnormalities [15]. However, cytologic samples taken during pregnancy (and up to 12 weeks postpartum) may be more difficult to interpret than samples taken at other times [20].

In Ukraine, cervical screening is recommended six-monthly for all women of childbearing age [21]. It is available free of charge at public health clinics, but is predominantly opportunistic. National policy includes a low-level recommendation (expert opinion) for screening as part of antenatal care, but there are no figures on coverage in this group or nationally [22]. World Health Survey data suggest that coverage with at least three-yearly screening in the general population is similar in Ukraine to many other European countries [23]. However, it is unclear whether this is the case among HIV positive women, who are more likely to be socially excluded [1] and require more intensive surveillance. Age-standardised mortality rates for cervical cancer are two-fold higher in Eastern than in Western Europe (7.1 vs 3.4 per 100,000) [24] and little is known about the prevalence of abnormal cervical cytology among HIV-positive women living in Eastern Europe.

We aimed to explore coverage of cervical screening as part of HIV care in a cohort of HIV-positive childbearing women in Ukraine receiving care at HIV/AIDS Centres, and to identify factors associated with an abnormal finding.

## Methods

The Ukraine Cohort Study of HIV-infected Childbearing Women (“Women’s Cohort”) is an ongoing study established in December 2007. HIV-infected women who had recently given birth (usually within the last 12 months) and were receiving care at one of five participating regional HIV/AIDS Centres in Ukraine (situated in Odessa, Donetsk, Kiev, Kriviy Rig and Mykolaiv) were enrolled with informed consent [25]. This postnatal cohort is nested within the European Collaborative Study (ECS), a birth cohort study in which pregnant HIV-infected women in ten European countries are enrolled, and their infants prospectively followed according to a standard protocol [26]. The Ukraine ECS has been enrolling women since 2000 [27] and around 80% of women in the postnatal study were also in the ECS, allowing linkage across the two studies (both collect coded anonymised data with unique identifiers).

Maternal socio-demographic information was collected as part of the ECS (clinician-completed questionnaire) and at enrolment in the Women’s Cohort (woman-completed questionnaire). Clinical information, including data on cervical screening, was provided by the clinician at enrolment and thereafter when a woman returned to the HIV/AIDS Centre for care. Data collection was opportunistic and dependent on routine clinic visits; according to policy, clinic visits should be at least three monthly for women on ART and six-monthly for those not on treatment, but in practice follow-up intervals could be much longer. All clinicians providing care at the HIV/AIDS Centres were gynaecologists. Only information on screening tests received as part of HIV care was available to them and reported in this study.

### Definitions

Results of cervical cytology were reported according to the 2001 Bethesda System as negative for intraepithelial lesion or malignancy (‘normal’), low-grade squamous intraepithelial lesion (LSIL) (corresponding to HPV infection, mild dysplasia or cervical intraepithelial neoplasia (CIN) 1) and high-grade squamous intraepithelial lesion (HSIL) (corresponding to moderate and severe dysplasia, carcinoma in situ or CIN 2 and CIN 3) [28]. A finding of LSIL or HSIL was defined as an abnormal result.

History of IDU was classified by self-report, clinical assessment or abstinence syndrome in the neonate. HIV clinical status was defined according to the World Health Organization (WHO) clinical staging criteria (advanced and severe symptomatic HIV disease corresponding to WHO stages 3 and 4 [29]). Previous pregnancy was defined as a previous live birth, stillbirth, miscarriage or termination, and multiparity as ≥1 previous live or stillbirth. Age at enrolment was categorised approximately into quartiles (16–23, 24–26, 27–30 and ≥31 years). Affordability of contraception was based on self-report.

Genital infections diagnosed with the following methods during the most recent pregnancy or postnatally (up to date of enrolment) were reported: bacterial vaginosis (BV) by Gram stain microscopy in 99% and by symptoms in 1%; vulvo-vaginal candida by Gram stain microscopy in 77%, culture in 14% and symptoms in 10%; *Chlamydia Trachomatis* (‘chlamydia’) by enzyme immunoassay on endocervical swab; *Trichomonas vaginalis* (TV) by microscopy.

### Data Analysis

Of the five centres participating in the study, analyses were limited to Odessa, Kiev and Donetsk, as Kriviy Rig and Mykolaiv did not report any cervical screening.

The χ^2^ and Fisher’s exact tests for categorical variables were used to investigate univariable comparisons. Univariable and multivariable Poisson regression models with a robust variance estimate were fitted to estimate unadjusted and adjusted prevalence ratios (PRs and APRs) of having a cervical screening test reported at postnatal study enrolment, and of an abnormal finding (LSIL or HSIL). The models for having a test reported were fitted including all women and also restricted to those diagnosed prior to conception of their most recent pregnancy. The PRs and APRs thus estimated provide a more interpretable measure of effect than those resulting from odds ratios obtained with logistic regression models, which may be inflated where outcomes are common, and control for under-dispersion and confounding which depends on the measure of effect [30]. Explanatory variables investigated included socio-demographic factors (age, parity, educational status), health behaviours (alcohol use, smoking, IDU, contraceptive use, disclosure of HIV status), clinical status (WHO stage, CD4 count), use of postnatal ART, time since HIV diagnosis and self-reported affordability of contraception and coinfection with viral hepatitis. Study centre and year of enrolment (December 2007–08, 2009, 2010–11) were included a priori in the multivariable analyses of factors associated with having a test reported to account for differences in local policy and changing clinical practice over time. Variables which remained significantly associated with the outcome after these adjustments (Wald test *p*<0.1) were included in the multivariable models. In analyses of factors associated with an abnormal finding, all variables known to be associated with invasive cervical cancer and available in our dataset were included a priori in the models (use of oral hormonal contraception, smoking, parity, Herpes simplex virus 2 (HSV-2) infection, chlamydia infection and CD4 count) [31]. Other variables were considered for inclusion in the multivariable model only if significant in univariable analyses (*p*<0.10).

Sexually transmitted infections (STIs) and genital infections (vulvo-vaginal candida and BV) were not considered in the analyses of factors associated with having a cervical screening test reported due to problems with interpretation of potential associations, particularly in terms of direction of effect, e.g. cervical screening could have prompted investigation for infections and vice versa. However, associations between STIs/genital infections and cervical abnormality were explored as some infections are known risk factors for invasive cervical cancer.

At the time of analysis, only 21% (233/1120) of women had follow-up data available reflecting the recent establishment of this cohort, long follow-up intervals among women not receiving ART, and also possible loss to follow-up. As women with follow-up were not representative of the cohort as a whole with respect to health-seeking behaviours, follow-up data were omitted from analyses.

Data were managed in an Access 2003 database (Microsoft Corps, Redmond, WA, USA) and statistical analyses were performed in Stata version 11.0 (StataCorp LP, College Station, Texas, USA).

## Results

A total of 1120 women were enrolled at Odessa, Kiev and Donetsk centres from December 2007 to March 2011, at a median of 10 months postpartum (83% (924/1120) at ≥12 weeks postpartum). Median age at enrolment was 27.3 years (IQR 24.2, 30.6). HIV diagnosis occurred a median of 1.5 years (IQR 1.0, 2.5) prior to enrolment. For two-thirds (665/971), this was during their most recent pregnancy and for one third (306/971) prior to conception. Women diagnosed <6 months before enrolment (and therefore within the interval for cervical screening according to Ukraine policy) accounted for 7% (69/970).

Almost all (971/972) women were born in Ukraine and 23% (257/1120) had a history of IDU, about a third (89/255) of whom had a sex partner who also injected drugs. Of those without an IDU history, 12% (88/716) reported a sex partner who injected drugs. Overall a third (299/872) were seropositive for hepatitis C virus (HCV), 55% (163/299) of whom had an IDU history. A quarter (279/1111) did not know the HIV status of their partner, 39% (434/1111) were in a concordant partnership, 29% (317/1111) in a discordant partnership and 7% (81/1111) had no current partner. Of the 76% (851/1120) of women tested for chlamydia, 25% (215/851) were positive, of whom 78% (164/211) had at least one STI in addition to chlamydia and HIV (mostly HSV-2). Overall, 18% (197/1109) had advanced or severe symptomatic HIV disease (WHO stages 3 or 4), and 27% (255/959) had a CD4 count ≤350 cells/mm^3^. Almost half (44%, 422/949) were multiparous at enrolment. Of those who reported being sexually active postnatally, 88% (699/794) reported use of condoms, most (83%, 581/699) as their only method of contraception.

Cohort characteristics by cervical screening test receipt are shown in Table 1, and prevalence of coinfections diagnosed during pregnancy or postnatally in Table 2. Just under a third of women (337/1120) had a cervical screening test reported at enrolment. Over half of those with date of cervical screening test reported (180/310) had received their most recent test postnatally (median 28 weeks after delivery, 17% (30/180) <12 weeks after delivery), 24% (74/310) during pregnancy and 18% (56/310) pre-conception. Most (69%, 232/334) had been tested over six months and 34% (115/334) over one year previously (median 40 weeks prior to enrolment). Women diagnosed with HIV prior to conception were more likely than those diagnosed antenatally or intrapartum to have a screening test reported at enrolment (44% (136/306) versus 26% (176/665), χ^2^ = 31.06 *p*<0.01). Of the 783 women with no test at baseline, 22% (*n* = 176) had follow-up data available, of whom 39% (68/176) had been screened at follow-up.

**Table 1:** Cohort characteristics by cervical screening test report.

	Test reported at enrolment (*n* = 337)	No test at enrolment (*n* = 783)
**Median age at enrolment (IQR)**	28.0(25.2, 31.3)	27.0(23.7, 30.2)
**Marital status (** ***n*** ** = 1113)**		
Married	225 (67%)	412 (53%)
Cohabiting	73 (22%)	235 (30%)
Single†/widowed/divorced	38 (11%)	130 (17%)
**Previous pregnancies** ‡ **(** ***n*** ** = 903)**		
1	95 (32%)	287 (47%)
2	87 (29%)	147 (24%)
3 or more	116 (39%)	171 (28%)
**Age at leaving full-time education (** ***n*** ** = 653)**		
≤16 years	32 (14%)	71 (16%)
17–18 years	44 (20%)	84 (19%)
≥19 years	146 (66%)	276 (64%)
**History of injecting drug use (** ***n*** ** = 1120)**		
No	247 (73%)	616 (79%)
Yes	90 (27%)	167 (21%)
**Alcohol use postnatally (** ***n*** ** = 1104)**		
No	268 (81%)	603 (78%)
Yes	63 (19%)	170 (22%)
**History of smoking (** ***n*** ** = 1114)**		
No	98 (29%)	238 (31%)
Yes	236 (71%)	542 (69%)
**Current smoking (** ***n*** ** = 1111)**		
No	153 (46%)	389 (50%)
Yes	181 (54%)	388 (50%)
**Disclosure of HIV status to anyone (** ***n*** ** = 1120)**		
No	15 (4%)	42 (5%)
Yes	322 (96%)	741 (95%)
**Disclosure of HIV status to family/friends (** ***n*** ** = 1120)**		
No	107 (32%)	293 (37%)
Yes	230 (68%)	490 (63%)
**Disclosure of HIV status to partner (** ***n*** ** = 1120)**		
No	67 (20%)	189 (24%)
Yes	270 (80%)	594 (76%)
**WHO stage (** ***n*** ** = 1109)**		
1–2	255 (76%)	657 (85%)
3–4	82 (24%)	115 (15%)
**CD4 count (** ***n*** ** = 959)**		
≤ 200 cells/mm^3^	22 (7%)	63 (10%)
201–350 cells/mm^3^	56 (18%)	114 (18%)
> 350 cells/mm^3^	234 (75%)	470 (73%)
Median	468 cells/mm^3^	456 cells/mm^3^
**Taking ART postnatally (** ***n*** ** = 1114)**		
No	262 (78%)	643 (82%)
Yes	72 (22%)	137 (18%)
**Any OC** ‡ **use reported postnatally (** ***n*** ** = 1120)**		
No	291 (86%)	715 (91%)
Yes	46 (14%)	68 (9%)
**Can afford family planning (self-report) (** ***n*** ** = 1089)**		
No	40 (12%)	179 (24%)
Yes	292 (88%)	578 (76%)

† Includes non-cohabiting partnerships;

‡ Previous pregnancies include still births, live births, miscarriages and terminations. OC, oral hormonal contraceptive.

### Factors Associated with Having a Cervical Screening Test Result Reported as Part of HIV Care

There was no significant change over time in the proportion of women with a screening test reported as part of HIV care at study enrolment (30% overall, *p* = 0.87). In univariable analyses, age, marital status, number of previous pregnancies, IDU history, WHO clinical stage, timing of HIV diagnosis, affordability of contraception and centre were significantly associated with having a screening test at the HIV/AIDS Centre (Table 3). HCV seropositivity and IDU history were both associated with report of a screening test in univariable analysis (χ^2^ = 9.69, *p*<0.01 and χ^2^ = 3.85, *p* = 0.05 respectively), but not on adjusting for year and centre (*p* = 0.44 and *p* = 0.48 respectively), and were therefore excluded from the multivariable model. HCV seropositivity is omitted from Table 3 due to its overlap with IDU history. Other factors that were not associated with having a test reported were smoking (current or history), current alcohol use, disclosure of HIV status to a partner, postnatal ART receipt and CD4 count.

**Table 2:** Coinfections by cervical screening test report at enrolment.

	Test reported at enrolment (*n* = 337)	No test at enrolment (*n* = 783)
Chlamydia (*n* = 851)	71 (25%)	144 (25%)
Syphilis (*n* = 907)	13 (4%)	17 (3%)
*Trichomonas vaginalis* (*n* = 645)	19 (6%)	52 (15%)
HSV-2 antibodies (*n* = 806)	156 (55%)	255 (49%)
Vulvo-vaginal candida (*n* = 739)	134 (43%)	232 (54%)
Bacterial vaginosis (*n* = 731)	67 (22%)	59 (14%)
Hepatitis C seropositive (*n* = 872)	120 (41%)	179 (31%)
Hepatitis B surface antigen positive (*n* = 1002)	23 (7%)	61 (9%)

In the multivariable model, there were significant differences in reporting of cervical screening tests by centre (Wald test *p*<0.01) (Table 3), and women were twice as likely to have a test reported if diagnosed prior to their most recent pregnancy rather during the 3^rd^ trimester or intrapartum. Women with one previous pregnancy (vs ≥2) were less likely to have a test reported (*p* = 0.05).

**Table 3:** Factors associated with having a cervical screening test reported at study enrolment.

	Proportion (*n*) with test reported at enrolment	Crude PR (95% CI) *n* = 870†	*p-*value	Adjusted PR – multivariable model (95% CI) *n* = 870	*p-*value
**Age at enrolment**					
16–23 years	21% (55/265)	1.00		1.00	
24–26 years	31% (78/255)	1.25 (0.91,1.73)	0.17	1.07 (0.78,1.47)	0.68
27–30 years	33% (111/339)	1.49 (1.11,2.00)	<0.01	1.13 (0.84,1.51)	0.42
≥31 years	36% (92/257)	1.55 (1.14,2.10)	<0.01	1.24 (0.90,1.69)	0.18
**Marital status**					
Married	35% (225/637)	1.00		1.00	
Cohabiting	24% (73/308)	0.72 (0.56,0.92)	<0.01	0.81 (0.63,1.04)	0.10
Single/widowed/divorced	23% (38/168)	0.68 (0.50,0.93)	0.02	0.84 (0.61,1.16)	0.29
**Previous pregnancies at enrolment**					
≥2	39% (203/521)	1.00		1.00	
1	25% (95/382)	0.64 (0.52,0.79)	<0.01	0.80 (0.65,1.00)	0.05
**History of IDU**					
No	29% (247/863)	1.00			
Yes	35% (90/257)	1.24 (1.01,1.52)	0.04		
**WHO clinical stage**					
1–2	28% (255/912)	1.00		1.00	
3–4	42% (82/197)	1.37 (1.12,1.68)	<0.01	1.07 (0.87,1.32)	0.51
**Timing of HIV diagnosis**					
Prior to conception	44% (136/306)	1.00		1.00	
1^st^/2^nd^ trimesters	28% (155/545)	0.60 (0.50,0.73)	<0.01	0.62 (0.51,0.75)	<0.01
3^rd^ trimester/intrapartum	18% (21/120)	0.41 (0.27,0.61)	<0.01	0.42 (0.28,0.63)	<0.01
**Affordability of contraception**					
Can afford	34% (292/870)	1.00		1.00	
Can’t afford	18% (40/219)	0.62 (0.45,0.85)	<0.01	0.76 (0.54,1.07)	0.12
**Year of enrolment**					
2007/08	30% (101/339)	1.00		1.00	
2009	31% (135/435)	1.05 (0.84,1.31)	0.67	0.96 (0.76,1.20)	0.71
2010/11	29% (101/344)	1.18 (0.93,1.50)	0.17	0.97 (0.76,1.23)	0.80
**Centre of enrolment**					
Odessa	26% (111/419)	1.00		1.00	
Kiev	39% (207/528)	1.26 (1.03,1.55)	0.03	1.32 (0.07,1.63)	<0.01
Donetsk	11% (19/173)	0.42 (0.25,0.71)	<0.01	0.49 (0.29,0.82)	<0.01

† Limited to 870 women included in the multivariable model.

In order to investigate factors associated with being screened at the HIV/AIDS Centre among women with longest exposure to HIV care, a sub-analysis was conducted limited to the 306 women diagnosed with HIV before their most recent pregnancy. In this group, 44% (136/306) of whom had a cervical screening test result reported at enrolment, age, marital status, self-reported affordability of contraception, HIV disclosure and centre were significantly associated with reporting of a test result in univariable analyses (Table 4). In the multivariable analyses adjusting for year, centre and affordability of contraception, women leaving full-time education at ≤16 (vs ≥19 years) and who were cohabiting (vs married) were less likely to have a test reported, although the latter was not statistically significant (*p* = 0.06) (Table 4). The four-category age variable did not significantly contribute to fit of the model when adjusting for year and centre (Wald test *p* = 0.14), however women ≥27 years were significantly more likely to have a test reported than those <27 years when a binary variable was used (APR 1.57, 95% CI 1.11–2.20 adjusting for marital status, education, affordability of contraception, year and centre, *p* = 0.01).

**Table 4:** Factors associated with having a cervical screening test reported at study enrolment, among women diagnosed with HIV prior to most recent pregnancy.

	Proportion (*n*) with test reported at enrolment	Crude PR *n* = 207†	*p*-value	Adjusted PR – multivariable model (95% CI) *n* = 207	*p-* value
**Age at enrolment**					
16–23 years	27% (12/44)	1.00			
24–26 years	39% (24/62)	1.22 (0.62,2.41)	0.56		
27–30 years	51% (56/110)	1.77 (0.98,3.21)	0.06		
≥31 years	49% (44/89)	1.93 (1.06,3.50)	0.03		
**Marital status**					
Married	55% (99/180)	1.00		1.00	
Cohabiting	30% (24/80)	0.41 (0.23,0.73)	<0.01	0.58 (0.32,1.03)	0.06
Single/widowed/divorced	27% (12/44)	0.55 (0.31,0.97)	0.04	0.74 (0.42,1.31)	0.30
**Age at leaving full-time education** ‡					
≥19 years	50% (60/121)	1.00		1.00	
17–18 years	46% (21/46)	0.94 (0.65,1.35)	0.75	0.79 (0.56,1.13)	0.20
≤16 years	36% (17/47)	0.73 (0.48,1.11)	0.15	0.66 (0.44,1.01)	0.05
**Self-reported affordability of contraception**					
Can afford	51% (114/223)	1.00		1.00	
Can’t afford	25% (19/75)	0.43 (0.25,0.74)	<0.01	0.79 (0.44,1.43)	0.44
**HIV status disclosure to family or friends**					
Yes	51% (95/188)	1.00			
No	35% (41/118)	0.61 (0.42,0.89)	0.01		
**WHO stage**					
1–2	41% (82/202)	1.00			
3–4	53% (54/101)	1.32 (0.99,1.77)	0.06		
**Year of enrolment**					
2007/08	38% (36/95)	1.00		1.00	
2009	45% (44/98)	1.08 (0.72,1.60)	0.72	0.85 (0.58,1.24)	0.39
2010/11	50% (56/112)	1.33 (0.91,1.93)	0.14	0.91 (0.61,1.36)	0.66
**Centre of enrolment**					
Odessa	48% (49/102)	1.00		1.00	
Kiev	56% (78/139)	1.27 (0.87,1.85)	0.22	1.11 (0.73,1.69)	0.63
Donetsk	14% (9/65)	0.39 (0.19,0.79)	0.01	0.38 (0.18,0.80)	0.01

† Limited to the 207 women included in the multivariable model.

‡ Significant on adjusting for year and centre (LRT *p* = 0.01) and thus included in the multivariable model, as specified in the methods.

Although not considered in Poisson regression analyses for reasons specified in the methods, BV was more common among women with a cervical screening test result reported than among those without (22% (67/311) vs 14% (59/420) respectively, χ^2^ = 7.04, p<0.01). Women with a cervical screening test reported were also more likely to have been tested for BV (92%, 311/337 vs 54% of those without a screening test, 420/783, χ^2^ = 155.21, *p*<0.01) and HSV-2 antibodies (85% (285/337) vs. 67% (521/783) of those without a screening test, χ^2^ = 37.96, *p*<0.01).

### Cervical Abnormalities

At enrolment, among the 30% with a screening test result reported, prevalence of cervical abnormalities at the most recent test was 21% (68/325) overall (17% (*n = *54) LSIL and 4% (*n* = 14) HSIL). Results were not available for 4% (12/337) of those tested, presumably because the sample was inadequate. In total, 38% (123/325) of women with a screening test result reported at enrolment had the test conducted on the same day as a positive sample was taken or diagnosis made for at least one of: chlamydia, gonorrhoea, syphilis, HSV-2, candida, TV or BV. Among the 68 women who only had a test reported at follow-up, prevalence of cervical abnormalities was 31% (21/68).

In crude analyses, women with BV infection were more likely to have a diagnosis of LSIL or HSIL, as were those with two or more previous pregnancies and those who were HSV-2 seropositive (Table 5). No other factors were significantly associated with abnormal findings. In the multivariable model, HSV-2 seropositivity was associated with an 83% increased risk of an abnormal finding and BV diagnosed antenatally or postnatally with over a three-fold increased risk (Table 5).

**Table 5:** Factors associated with an abnormal finding (LSIL or HSIL) on cervical screening, among women with a test reported at study enrolment.

	Proportion (*n*) with abnormal result	Crude PR (95% CI) *n* = 213†	*p-*value	Adjusted PR‡ (95% CI) *n* = 213	*p-*value
**Age at enrolment**					
16–23 years	23% (15/65)	1.00		1.00	
24–26 years	23% (18/77)	1.60 (0.55–4.68)	0.39	1.52 (0.57–4.04)	0.41
27–30 years	24% (21/89)	1.97 (0.72–5.40)	0.19	1.72 (0.69–4.31)	0.24
≥31 years	20% (14/69)	1.81 (0.65–5.05)	0.26	1.42 (0.56–3.60)	0.46
**Previous pregnancies at enrolment**					
≥2	26% (51/195)	1.00		1.00	
1	15% (14/92)	0.44 (0.21–0.93)	0.03	0.56 (0.26–1.22)	0.15
**CD4 count**					
>350 cells/mm^3^	22% (50/228)	1.00		1.00	
201–350 cells/mm^3^	21% (11/53)	1.21 (0.65–2.27)	0.55	1.36 (0.74–2.49)	0.33
≤200 cells/mm^3^	25% (5/20)	1.57 (0.65–3.77)	0.31	2.07 (0.93–4.57)	0.07
**Currently smoking**					
No	25% (38/151)	1.00		1.00	
Yes	19% (33/171)	0.99 (0.59–1.67)	0.98	0.78 (0.49–1.26)	0.31
**Oral contraceptive use postnatally (any)**					
No	22% (61/279)	1.00		1.00	
Yes	24% (11/46)	0.94 (0.46–1.93)	0.86	0.95 (0.48–1.88)	0.88
**HSV-2**					
No	18% (22/125)	1.00		1.00	
Yes	27% (40/150)	1.73 (1.00–3.00)	0.05	1.83 (1.07–3.11)	0.03
**Chlamydia**					
No	20% (41/205)	1.00		1.00	
Yes	25% (17/68)	1.32 (0.75–2.32)	0.33	0.79 (0.46–1.36)	0.40
**Bacterial vaginosis**					
No	17% (40/236)	1.00		1.00	
Yes	39% (25/64)	3.36 (2.07–5.45)	<0.01	3.49 (2.11–5.76)	<0.01
***Trichonomas vaginalis***					
No	19% (52/269)	1.00			
Yes	44% (8/18)	1.80 (0.76–4.24)	0.18		

† Limited to 213 women included in the multivariable model.

‡ Adjusted a priori for age, previous pregnancies, CD4 count, current smoking, oral contraceptive use, HSV-2 and chlamydia and additionally for BV.

## Discussion

At enrolment in this postnatal cohort, only 30% of women had a cervical screening test reported as part of HIV care, a third of whom had been screened more than a year previously. Women diagnosed with HIV prior to rather than during their most recent pregnancy, and therefore with more opportunity to receive HIV care, were more likely to have a cervical screening test reported at enrolment, as were those with more previous pregnancies. A fifth of those screened had a finding of LSIL or HSIL.

In this study, we could only assess the coverage of cervical screening carried out as part of HIV care; women may have accessed screening through contraceptive, sexual health or other services. Screening is recommended six-monthly for the general population, but it is unclear whether this policy is followed in practice - in the 2003 World Health Survey 66% of 1361 women reported being screened in the last three years [22], but there were no data on screening frequency, or laboratory or clinical data, with which to validate self-reports. In addition to more regular screening, HIV-positive women may also benefit from more intensive follow-up following a mild abnormal smear, a lower threshold for referral to colposcopy (especially if severely immunosuppressed) and more intensive surveillance immediately following HIV diagnosis compared with the standard of care [17], [18]. However, this can only be offered if the healthcare provider is aware of the woman’s HIV status. Of those diagnosed as HIV-positive during their most recent pregnancy, only a quarter had been screened as part of HIV care by enrolment despite most having returned to the HIV/AIDS Centre at >12 weeks postpartum. As there is no policy for referral of newly diagnosed women to other services, it is likely that many did not receive any cervical screening in the year following HIV diagnosis. Even among the fifth of women with follow-up, coverage of cervical screening among those with no test reported at enrolment was only 39%.

The 30% of women with screening test results available in this study were a selected group whose characteristics (e.g. higher prevalence of BV) may mean findings are not generalisable to the cohort as a whole. Nevertheless, the 21% prevalence of abnormal findings on cytologic screening was comparable to the 23% prevalence of LSIL/HISL among 285 HIV-positive women recruited from a patient programme in Brooklyn from 1990–93 [32], and 27% prevalence of abnormalities reported among 1134 HIV-positive women enrolled at the European sites of a multi-site cohort study [33]. A study of 200 HIV-positive women attending mother-child health clinics in Zimbabwe, a country with postnatal opportunistic testing but no national screening policy, found a prevalence of cervical dyskaryosis of 30% [34]. In another study, prevalence of LSIL/HSIL among 400 HIV-positive women in South Africa was found to be 48% [33]. In the Women’s Interagency HIV Study, a large representative US study of HIV-positive women, the prevalence of LSIL/HSIL was lower at 15%, possibly because all women participated in six-monthly screening [35]. The role of ART in prevention of HPV-related cervical lesions or promotion of their regression is unclear [36]–[40]. With further roll-out of ART (currently available to only around half of adults with advanced HIV disease in Ukraine [2]) and decline in deaths due to other AIDS-defining diseases, the proportion of deaths attributable to cervical cancer may increase [41], particularly as the HIV-positive population ages.

There was a high prevalence of a number of co-factors implicated in the aetiology of cervical cancer in this cohort, including smoking, chlamydia and HSV-2 infections [31]. In adjusted analyses, HSV-2 seropositivity was associated with an 80% increased risk of LSIL/HSIL and BV with over a three-fold increased risk. These associations could have been due to selective screening of women at high risk for both HPV infection and infection with HSV-2 or BV (e.g. women with multiple sexual partners). However, co-infections to HPV may increase the risk of cervical cancer due to the effect of reactive oxidative metabolites generated by inflammatory processes local to the cervix [42], or by acting as cofactors [42]. A pooled analysis of seven studies found an increased risk of invasive cervical cancer associated with HSV-2 seropositivity independent of HPV infection and sexual risk behaviours [43]. Evidence of an association between BV and HPV acquisition/persistence or cervical abnormalities is less well established [44]–[49], but a recent meta-analysis of twelve studies (only three of which independently showed an association between BV and cervical HPV infection) demonstrated a significantly increased risk of cervical HPV infection among women with BV (combined OR 1.43, 95% CI 1.11–1.84) [50]. Two studies including HIV-positive women (one of which was included in the meta-analysis) showed an association between BV and both incident and prevalent cervical HPV infection, independent of sexual risk factors [51], [52]. Given the prevalence of BV in this cohort (17%), and the associated risk of other adverse effects (including preterm delivery [53] and STI acquisition [54]), regular screening for BV with prompt treatment where indicated should be a priority.

Women in poorer socioeconomic groups are less likely to be screened for cervical abnormalities in the US [55], and in Ukraine, where World Household Survey data showed coverage with three-yearly screening of 87% in the top and 68% in the bottom wealth quintiles [23]. HIV-positive women may be socially disadvantaged due to their poor health, discriminatory employment practices and coexisting behaviours (e.g. IDU). In our study, a fifth reported being unable to afford contraception and a third had not disclosed their HIV status to a parent, family member or friend, indicating both economic and social marginalisation. Among those diagnosed prior to conception, women with fewest years of education were least likely to have been screened. An organised screening programme could improve awareness and uptake among the most marginalised women. At an HIV clinic in the United Kingdom, a higher uptake of cervical screening was found among women on HAART compared with those not yet on treatment, probably due to their on-going engagement with HIV care [56]. Regular invitations to attend the HIV/AIDS Centre for screening could help prevent postpartum loss to follow-up among HIV-positive women not on ART. As national policy, organised screening programmes delivered as part of HIV care could also lessen the regional disparities in screening coverage.

In Eastern Europe 70% of cervical cancer cases are attributed to vaccine-preventable HPV types 16 and 18 [22], [57], but HPV vaccination programmes have yet to be introduced in Ukraine [22]. The safety and efficacy of HPV vaccination in immune-compromised populations have not yet been established [37]. Although an important future intervention, HPV vaccination will not obviate the need for an organised cervical screening programme in Ukraine.

### Limitations

Lack of data on cervical HPV infection or sexual risk behaviours precluded more detailed exploration of the association between BV and LSIL/HSIL. False positives or negatives cannot be ruled out, particularly as a quarter of the samples were taken in pregnancy, 10% at <12 weeks postpartum and 38% on the same day as a positive sample for a genital infection [20]. We are not able to comment on sensitivity and specificity of cytologic screening in this population, as colposcopy and histology takes place at referral hospitals and data are not routinely shared with the HIV/AIDS Centre. Because women with a screening test had a higher prevalence of BV than those without, the observed prevalence of cervical abnormalities in this study could be an overestimate. Furthermore, since cervical screening test results were only available for 30% of women, selection bias in the association between BV and cervical abnormalities (e.g. due to sexual risk-taking behaviour) cannot be ruled out. Local differences exist in provision of cervical screening services both within and outside of HIV care, and our results may therefore not be generalizable to other areas in Ukraine. Finally, coverage of cervical screening as part of HIV care may be higher in this cohort than in the wider population of HIV-positive women in Ukraine, as all women in the cohort were in contact with HIV healthcare services.

In conclusion, cervical screening coverage of this high risk population as part of HIV care is low. An organised programme where women are invited to attend the HIV/AIDS Centre for cervical screening could increase coverage, particularly among marginalised women. BV testing and treatment could potentially reduce vulnerability to cervical abnormalities.
